# Gender inequalities and the burden of anorexia and bulimia in Europe, 1990–2023: findings from the Global Burden of Disease Study 2023

**DOI:** 10.1192/j.eurpsy.2026.11378

**Published:** 2026-07-31

**Authors:** G. Castelpietra, L. Monasta, G. Zamagni

**Affiliations:** 1 Centre Neuchatelois Psychiatrie, Marin-Epagnier, Switzerland; 2 Institute for Maternal and Child Health-IRCCS “Burlo Garofolo”; 3 Institute for Maternal and Child Health-IRCCS “Burlo Garofolo, Trieste, Italy

## Abstract

**Introduction:**

Eating disorders (EDs), including anorexia nervosa (AN) and bulimia nervosa (BN), are severe mental health conditions affecting physical and psychosocial health. Structural gender inequality may shape the burden of EDs across sexes and regions.

**Objectives:**

We aimed to quantify the burden of EDs among young males and females in Europe using GBD 2023 estimates and to explore its association with the Gender Inequality Index (GII). Quantifying the burden of EDs in terms of YLDs and examining their implications for health systems can generate essential evidence to guide public health priorities, improve recognition and care, and foster more equitable and effective responses

**Methods:**

We analyzed Global Burden of Disease Study 2023 estimates to assess the relationship between gender inequality and ED burden in Europe. Years Lived with Disability (YLD) for AN and BN were extracted by sex and five-year age groups (10–39 years) from 1990–2023 across 35 countries.

Gender inequality was measured using the GII. Mixed linear regression was applied with year, age group, sex, GII, and their interactions as fixed effects, and country and year as random intercept and slope, respectively.

**Results:**

GII showed considerable variation across Western, Central, and Eastern Europe in 2023, as did YLDs for AN and BN (Figure 1). YLDs of both AN and BN increased from 1990 to 2023 across most countries and age groups, with females consistently experiencing the highest burden (Figure 2 and 3). For AN, higher levels of GII were significantly associated with greater YLD rates in both males (β = 29.28; 95% CI: 22.2 to 36.4) and females (β = 85.3; 95% CI: 42.1 to 128.6), with a stronger effect for females. For BN, GII was inversely associated with YLDs among males (β = –645.6; 95% CI: –732.8 to –558.3), while a positive association was found in females (β = 22 794.7; 95% CI: 659.1 to 930.2).

**Image 1:**
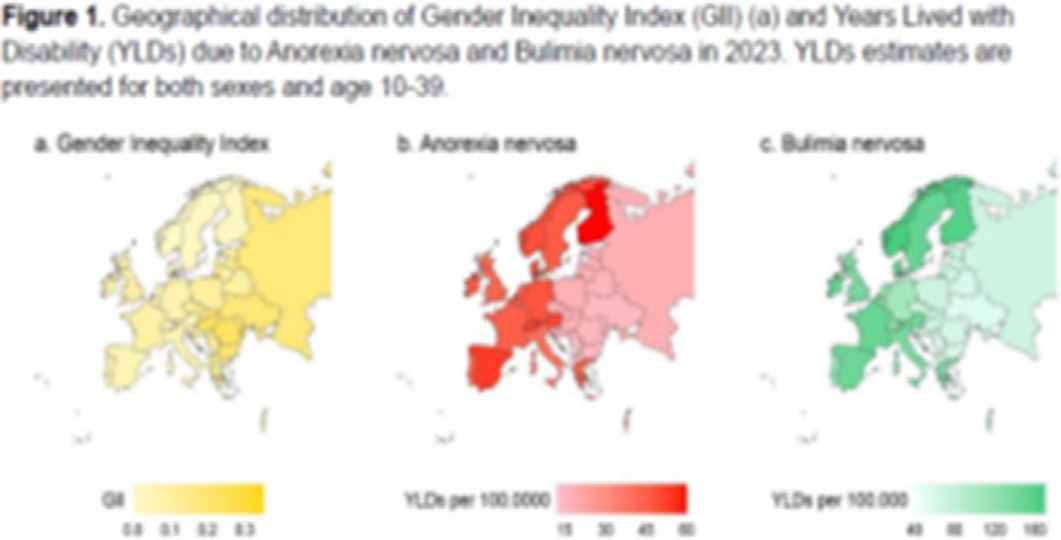
Image 1: Long description.

**Image 2:**
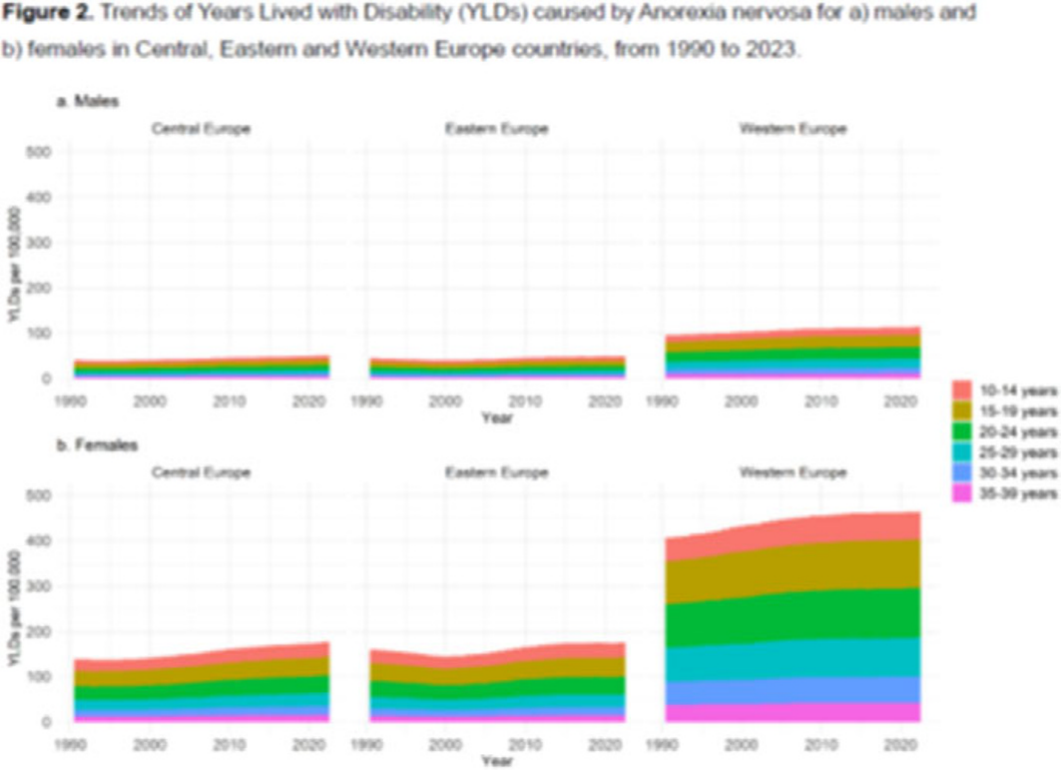
Image 2: Long description.

**Image 3:**
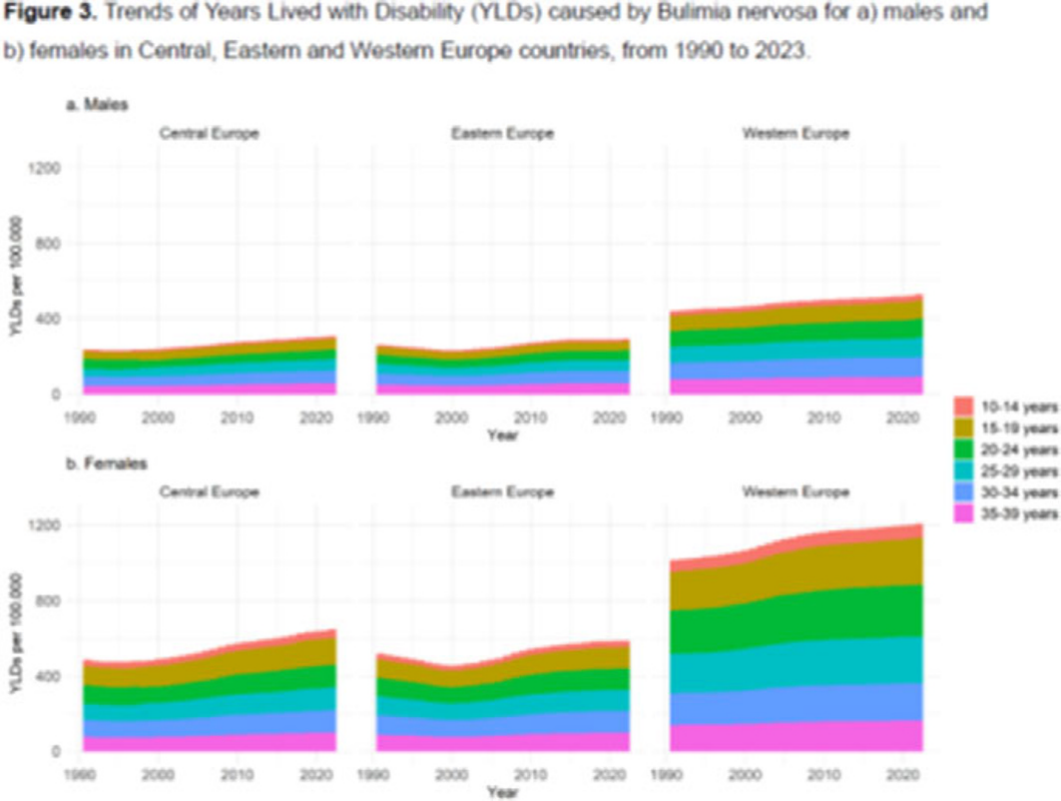
Image 3: Long description.

**Conclusions:**

Gender inequality is linked to contrasting patterns in ED burden among European adolescents and young adults. Results highlight the need for gender-sensitive mental health strategies, particularly in settings marked by high inequality

**Disclosure of Interest:**

None Declared

## Long descriptions

### Image 1: Long description

A three-panel set of geographical maps focusing on Europe and parts of Western Asia.

Panel a, titled Gender Inequality Index, uses a yellow gradient scale for G I I from 0.0 to 0.3. The highest inequality values are concentrated in the Southeast and Eastern regions, while Northern and Western Europe show the lowest values in pale yellow.

Panel b, titled Anorexia nervosa, displays Y L D s per 100,000 using a red gradient scale from 15 to 60. The highest density of years lived with disability is concentrated in Northern Europe and the Iberian Peninsula in deep red, with lower concentrations in Eastern Europe and Western Asia in pale pink.

Panel c, titled Bulimia nervosa, displays Y L D s per 100,000 using a green gradient scale from 40 to 160. Similar to the anorexia map, the highest values are concentrated in Northern and Western Europe in dark green, while the Southeast and Eastern regions show significantly lower values in pale green.

Navigate back to Image 1:.

### Image 2: Long description

The chart consists of two rows of three panels each. The top row (a) represents Males and the bottom row (b) represents Females. Each row contains panels for Central Europe, Eastern Europe, and Western Europe. The x-axis represents the Year from 1990 to 2023, and the y-axis represents Y L Ds per 100,000, ranging from 0 to 500.

Data is displayed as stacked area charts categorized by age groups:

* 10-14 years (pink, top layer)

* 15-19 years (olive green)

* 20-24 years (bright green)

* 25-29 years (teal)

* 30-34 years (light blue)

* 35-39 years (magenta, bottom layer)

Row a (Males):

* Central and Eastern Europe: Trends are nearly flat and low, remaining below 50 Y L Ds per 100,000.

* Western Europe: Shows a slight linear increase over time, reaching approximately 100 Y L Ds per 100,000 by 2023.

Row b (Females):

* Central Europe: Shows a steady linear increase from approximately 140 in 1990 to 180 Y L Ds per 100,000 in 2023.

* Eastern Europe: Displays a slight dip around the year 2000 before rising back to approximately 180 Y L Ds per 100,000 by 2023.

* Western Europe: Shows the highest values and steepest growth, starting at 400 in 1990 and rising to nearly 480 Y L Ds per 100,000 by 2023. The 15-19 and 20-24 age groups constitute the largest portions of the total Y L Ds in this region.

Navigate back to Image 2:.

### Image 3: Long description

A multi-panel figure with two rows and three columns. The Y-axis represents Y L D s per 100,000 ranging from 0 to 1200. The X-axis represents Year from 1990 to 2020. A legend on the right identifies six age groups as stacked colored layers: 10-14 years (red), 15-19 years (olive), 20-24 years (green), 25-29 years (teal), 30-34 years (blue), and 35-39 years (pink).

Row a. Males:

* Central Europe: Shows a gradual linear increase from approximately 200 to 300 total Y L D s.

* Eastern Europe: Displays a slight dip around the year 2000 before rising back to approximately 300 Y L D s by 2023.

* Western Europe: Shows the highest values for males, starting near 400 and rising steadily to over 500 Y L D s.

Row b. Females:

* Central Europe: Starts at approximately 500 and increases to nearly 700 Y L D s.

* Eastern Europe: Shows a U-shaped curve with a minimum around 2000, ending near 600 Y L D s.

* Western Europe: Displays the highest overall values in the figure, starting at 1000 in 1990 and rising to approximately 1200 Y L D s by 2023.

In all panels, the 15-19 and 20-24 age groups (olive and green) represent the largest portions of the stacked area, indicating these ages contribute most to the Y L D s.

Navigate back to Image 3:.

